# Russian Gargle

**Published:** 1879-12

**Authors:** 


Russian Gargle.—Carbolic acid and tannic acid, each fifteen parts; alcohol, sixty parts; distilled water, one hundred and twenty parts. A teaspoonful of this is added to half a pint of water in order to form a gargle. This solution is largely employed in Russia at the commencement of angina and in chronic inflammations of the throat.—*L'Union Med.; Med. Times and Gazette.*

